# HIF-1α Is a Rational Target for Future Ovarian Cancer Therapies

**DOI:** 10.3389/fonc.2021.785111

**Published:** 2021-12-24

**Authors:** Xin Wang, Zhen-wu Du, Tian-min Xu, Xiao-jun Wang, Wei Li, Jia-li Gao, Jing Li, He Zhu

**Affiliations:** ^1^ Department of Obstetrics and Gynaecology, The Second Hospital of Jilin University, Changchun, China; ^2^ Department of Orthopaedics, The Second Hospital of Jilin University, Changchun, China; ^3^ Research Center, The Second Hospital of Jilin University, Changchun, China

**Keywords:** ovarian cancer, hypoxia-inducible factors, targeted therapy, molecular target, tumour microenvironment

## Abstract

Ovarian cancer is the eighth most commonly diagnosed cancer among women worldwide. Even with the development of novel drugs, nearly one-half of the patients with ovarian cancer die within five years of diagnosis. These situations indicate the need for novel therapeutic agents for ovarian cancer. Increasing evidence has shown that hypoxia-inducible factor-1α(HIF-1α) plays an important role in promoting malignant cell chemoresistance, tumour metastasis, angiogenesis, immunosuppression and intercellular interactions. The unique microenvironment, crosstalk and/or interaction between cells and other characteristics of ovarian cancer can influence therapeutic efficiency or promote the disease progression. Inhibition of the expression or activity of HIF-1α can directly or indirectly enhance the therapeutic responsiveness of tumour cells. Therefore, it is reasonable to consider HIF-1α as a potential therapeutic target for ovarian cancer. In this paper, we summarize the latest research on the role of HIF-1α and molecules which can inhibit HIF-1α expression directly or indirectly in ovarian cancer, and drug clinical trials about the HIF-1α inhibitors in ovarian cancer or other solid malignant tumours.

## Introduction

Ovarian cancer is the eighth most commonly diagnosed cancer among women worldwide (1). Epithelial ovarian cancer (EOC) represents one of the deadliest cancers among women, with 47% of patients dying 5 years after EOC diagnosis (2). The standard treatment for ovarian cancer is debulking surgery combined with chemotherapy (3). Unfortunately, even when patients accept standard treatment, recurrence occurs within 2 years in approximately 75% of patients who suffer from advanced-stage EOC (4).

The complex and rich multicellular environment in which a tumour develops is defined as the tumour microenvironment (TME) (5). In recent years, numerous studies have indicated that the TME plays a vital role in the malignant biological properties of tumours (6, 7), including ovarian cancer (8). With the uncontrolled growth of tumour cells and abnormalities in tumour microcirculation (9), hypoxia is an obvious feature of the TME, which is positively associated with tumour growth, angiogenesis, resistance to apoptosis and chemotherapy, and tumour metastasis (10). Hypoxia-inducible factors (HIFs) constitute a family of transcription factors that are involved in the regulation of the cellular response to hypoxic stress (11)and include three members: HIF-1 (12), HIF-2 (13), and HIF-3 (14).

**Figure 1:**
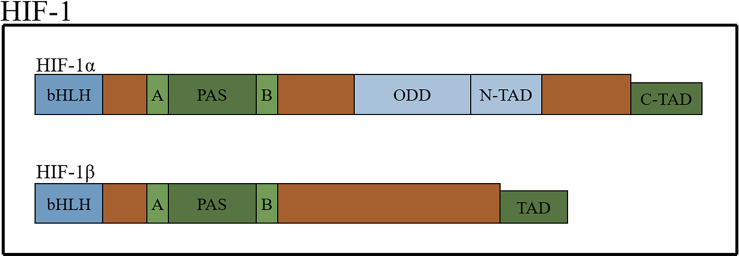
The schematic structure of HIF-1 protein.

HIFs, which form dimers, are composed of an oxygen-sensitive α-subunit and constitutively expressed β subunit (15, 16). There are three types of α-subunits (HIF-1α, HIF-2α and HIF-3α). The structures of HIF-1α and HIF-2α are similar but not identical, and they heterodimerize with the aryl hydrocarbon nuclear receptor translocator (also known as HIF-1β) to form HIF-1 and HIF-2, respectively (17). HIFs belong to the basic-helix-hoop-helix Per-Arnt-Sim (bHLH-PAS) protein family and contain a bHLH domain (the bHLH domain mediates the DNA-binding activity of HIF-α through the specific amino acids located in this domain), followed by a PAS domain. There are two different PAS domains, named PAS-A and PSA-B. The PAS domain of HIF-1α is required for the binding of hypoxia response elements (HREs) and the formation of active heterodimers. HIFs also contain an oxygen-dependent degradation domain (ODD) that is highly conserved and N-terminal and C-terminal transactivation domains (18–23) (**Figure 1**).

Numerous studies have found that HIF-1 participates in the process of metastasis, resistance to chemotherapy or radiotherapy and cancer stem-like cell maintenance in various types of cancers (24, 25) and is associated with the prognosis of gynaecological cancers (26). Thus, considering the constitutive expression of the β subunit, targeting HIF-1α may be a novel approach to treat ovarian cancer. This review summarizes recent studies on HIF-1α in ovarian cancer.

## HIF-1α Is Considered a Poor Prognostic Factor for Ovarian Cancer

The significance of HIF-1α in solid malignant cancer varies. It is a favourable prognostic factor in renal cell cancer and early-stage squamous cell carcinomas of the oral floor (34, 35), but unfavourable in breast or oesophageal squamous cell carcinoma (35, 36). Many studies have indicated that a shorter OS is related to the positive HIF-1α expression (30–32, 37, 38). In late-stage and poorly differentiated ovarian cancer, positive HIF-1α expression is related to a shorter OS time but not a shorter progression-free interval(PFI), while patients who underwent suboptimal cytoreduction and had positive HIF-1α expression exhibited a shorter PFI than HIF-1α-negative patients (29).Only one report found no association between HIF-1α and the overall survival (OS) of ovarian cancer (27). In summary, the majority of studies have indicated that HIF-1α is a good predictor of a poor prognosis in ovarian cancer (**Table 1**).

**Table 1:** The association between HIF-1α expression and clinical characteristics.

Ref.	Case	Method to evaluation HIF-1α	OS	DFS	PFI	PFS	Stage	LN-metastasis	Grade	Chemotherapy-sensitivity
(27)	102	IHC	P=0.183^a^	P=0.353^a^	(–)	(-)	P=0.468	(–)	P<0.001	P=0.885
			P=0.3950^b^	P=0.6848^b^						
(28)	52	WB	(–)	(–)	(–)	(–)	Not significant^d^	(–)	Not significant^d^	P<0.01^c^
(29)	55	IHC	P<0.01^a^	(–)	P>0.05	(–)	(–)	(–)	(–)	(–)
			P<0.01^e^		P<0.05^f^					
(30)	124	IHC	P=0.113^a^	(–)	(–)	P=0.113^a^	P=0.000	P=0.000	P=0.036	P=0.149
			P<0.000^b^			P=0.031^b^				
(31)	275	ELISA	P=0.009^a,i^	(–)	(–)	Not significant^d^	P=0.0896	(–)	P=0.152	P=1
(32)	76	IHC	P=0.003^e^	(–)	(–)	(–)	0.019	P=0.024	P=0.005	(–)
(33)	60	IHC	P=0.001^e^	(–)	(–)	(–)	0.007	P<0.001	P=0.006	P=0.022^j^

a Multivariate analysis;

b univariate analysis;

c higher HIF-1α expression indicates better chemotherapy sensitivity;

d p-value not shown;

e Kaplan-Meier survival curve analysis;

f positive HIF-1α expression indicated shorter PFI in patients undergoing suboptimal cytoreduction;

g all patients were stage III/IV;

h stage I was excluded;

i cut-off value of HIF-1α was 80 pg/mg;

j low HIF-1α expression was positively associated with a good response to chemotherapy.

HIF-1α expression may be associated with the response to chemotherapy. Alabiad et al. reported a good response to chemotherapy in patients with low HIF-1α expression (33). In contrast, researchers found that HIF-1α-expressing patients were more sensitive to paclitaxel/carboplatin combination chemotherapy (28), and Birner noted that HIF-1α does not influence the response to platinum-based chemotherapy (27). Considering the large number of cell experiments proving that HIF-1α contributes to the chemoresistance of ovarian cancer (discussed later) and the small number of samples in the studies mentioned previously, we need to further investigate the relationship between HIF-1α expression and chemotherapy sensitivity (**Table 1**).

## HIF-1α Promotes Ovarian Cancer Progression Through Several Biological Processes

### HIF-1α Can Inhibit the Function of p53

**Figure 2:**
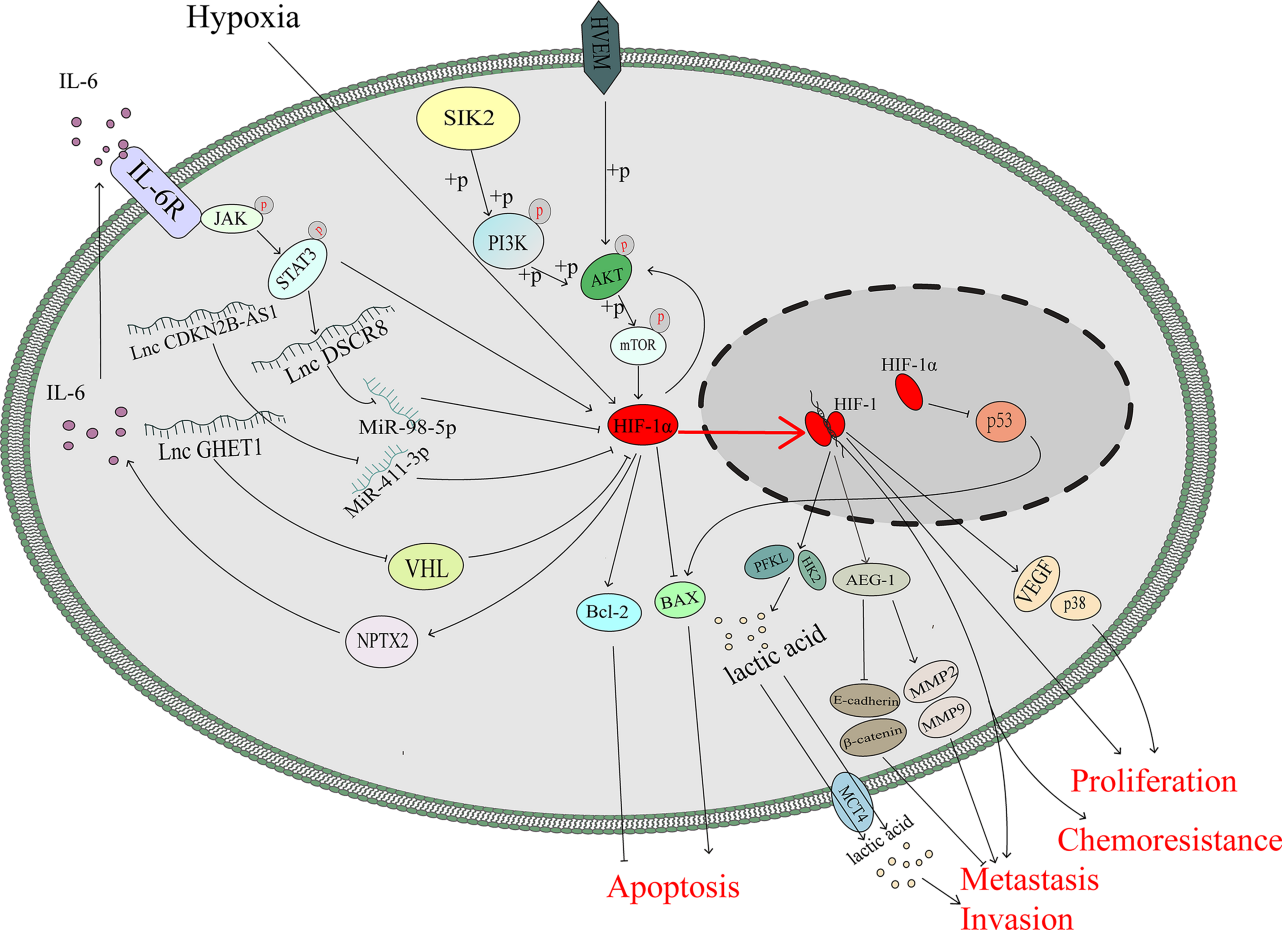
HIF-1α promotes ovarian cancer progression not only through several classical pathway but also through the function of p53 and changes in metabolism.

As an important tumour suppressor, p53 plays an important role in modulating drug sensitivity (39–41).After mimicking hypoxic stress, the p53 protein, which is supposed to be induced by doxorubicin or cisplatin, was downregulated so that apoptosis of lung and colon cancer cells mediated through p53 protein was diminished (42). Cisplatin can kill ovarian cancer cells through the p53-dependent apoptotic pathway (43, 44). Basmina et al. found that HIF-1α protein binding to p53 protein, so that the transcriptional function of p53 decreased, and thus the expression of BAX downregulated, thereby affecting the apoptosis process mediated by p53 (45). Scientists have already discovered that the ODD region of the HIF-1α protein can directly bind to the DNA-binding region of the p53 protein and may abolish the function of p53, thus hampering gene transactivation in nonmalignant cells (46).However, the accurate binding mechanism between the p53 protein and HIF-1α protein in ovarian cancer is still not clear (**Figure 2**).

### HIF-1α Promotes the Expression of IL-6

Interleukin-6 (IL-6) is a multifunctional cytokine that participates in the progression of many kinds of malignant tumours (47). IL-6 is highly expressed in the serum and ascites of patients with ovarian cancer, and its upregulation is significantly associated with the poor prognosis (48–50). In colon tumour cells, HIF-1α can regulate IL-6 expression *via* miR-338-5p (51).However, under hypoxic stress, the HIF-1 complex can promote the transcription and expression of neuronal pentraxin II (NPTX2). IL-6 expression is upregulated by NPTX2 overexpression, and the JAK2/STAT3 axis is activated *via* overexpression of IL-6 to promote the proliferation, invasion and migration of EOC cells (52). In addition, IL-6 can induce nuclear translocation and elevate the transcriptional activity of HIF-1α *via* STAT3 signalling to enhance the chemoresistance against cisplatin of ovarian cancer cells (53). It seems that there is a positive feedback loop between HIF-1 and IL-6 that is mediated by JAK/STATA3 signalling (**Figure 2**).

### LncRNAs Promote the Progression of Ovarian Cancer *via* HIF-1α

Long noncoding RNAs play variable roles in malignant tumours. HIF-1α can regulate the expression of these noncoding RNAs, and noncoding RNAs can interact with mRNA-HIF-1α to regulate the expression of HIF-1α protein and then induce the progression of many types of tumours, including breast cancer (54) and ovarian cancer (55, 56).

The lncRNA CDKN2B-AS1 is overexpressed in ovarian cancer and can silence miR-411-3p, release HIF-1α mRNA, whose translation production plays a critical role in the transcription of VEGF and p38, and then promote the migration and invasion of cancer cells (55). LncRNA DSCR8 is upregulated in ovarian cancer tissue and promoted tumour growth. HIF-1α promote the expression of DSCR8, which can sponge miR-98-5p, so that stopping miR-98-5p targeting to the 3’-UTR of STAT3 and then promoting ovarian cancer progression by stimulating the STAT3/HIF-1α pathway, which in turn upregulates DSCR8, creating a positive feedback loop to promote the progression of ovarian cancer (56) (**Figure 2**).

### HIF-1α Can Stimulate the AKT/mTOR Pathway

AKT/mTOR pathway plays a vital role in the progression of ovarian cancer (57). Knockdown the HIF-1α expression *via* siRNA in A2780 and SKOV3 cells significantly downregulated the phosphorylation of AKT/mTOR (58).Besides, AKT pathway regulates the expression of HIF-1α (59, 60) and Herpesvirus entry mediator(HVEM) is overexpressed in ovarian cancer (61). A hypoxic environment upregulates HVEM expression and enhances the phosphorylation of AKT/mTOR, thus inducing the expression of HIF-1α, which can promote the cell proliferation (62). It is speculated that the HEVM/AKT/mTOR/HIF-1α axis and HIF-1α/AKT/mTOR axis may construct a feedback loop to promote ovarian cancer progression, which needs further investigation (**Figure 2**).

### HIF-1α Promotes the Glycolysis Pathway in Ovarian Cancer

Metabolites of the glycolysis pathway are abnormally activated in malignant tumours even under normoxia (called the Warburg effect) and promote the progression of cancers (63), including gallbladder cancer (64), pancreatic cancer (65), cervical cancer (66) and ovarian cancer (67). HIF-1α, as a transcription factor, can regulate metabolism-associated genes, which contribute to Warburg effect (68, 69). SIK2 is associated with poor outcomes in ovarian cancer, and previous studies have demonstrated that SIK2 induces ovarian cancer progression by activating the PI3K/AKT pathway (70, 71). SIK2 upregulates the expression level of HIF-1α, which enhances the transcription of glycolysis-associated genes (HK2 and PFKL), inducing the metastasis and invasion of ovarian cancer (72). As the major rate-limiting enzymes in the glycolysis pathway, HK2 and PFKL overexpression promotes Warburg effect, which could assist the uncontrolled proliferation of cancer cells (73–75). The expression level of the long noncoding RNA (lncRNA) GEHT1 is enhanced in ovarian cancer tissue compared with normal tissue and is associated with poor prognosis. LncGEHT1 can interact with von Hippel-Lindau (VHL) to block the degradation of HIF-1α, thus modulating lactate production and influencing the growth of ovarian cancer (76) (**Figure 2**).

## Crosstalk Between Malignant Tumours and Nonmalignant Tumour Cells Mediated *via* HIF-1α Accelerates the Progression of Ovarian Cancer

### Mesothelial Cells

**Figure 3:**
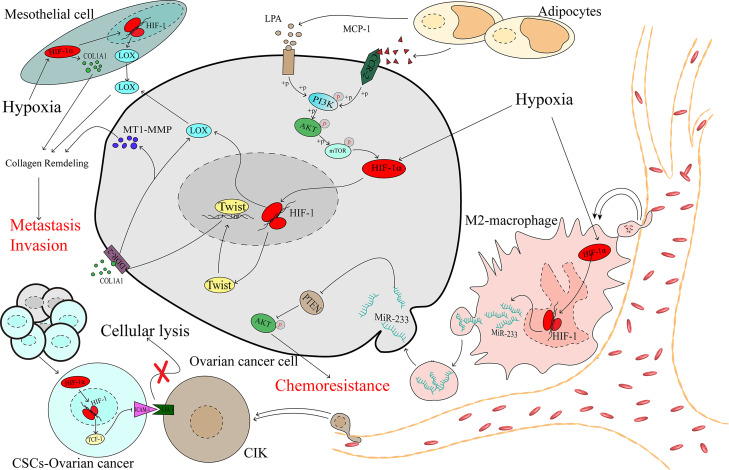
The complex microenvironment accelerates the development of ovarian cancer as mediated by HIF-1α.

Mesothelial cells are among the main cellular components compromising the peritoneal cavity and omentum, which are the most common metastatic sites of advanced ovarian cancer. Mesothelial cells have been proven to play a critical role in contributing to ovarian cancer metastasis (77). A collagen-remodelling gene signature containing COL1A1 and LOX is associated with the progression of ovarian cancer and unfavourable patient survival (78). Lysyl oxidase (LOX) has been proven to act as a tumour promoter (79) and regulate by HIF-1α in ovarian cancer (80). Under hypoxic stress, HIF-1 could promote the expression of COL1A1 in the mesothelial cells and the expression of LOX in both the mesothelial and cancer cells, which remodels collagen to accelerate the invasion of ovarian cancer (81) (**Figure 3**).

### Immune Cells

The tumour immune microenvironment contains immune cells that play considerable roles in the processes of tumour promotion and suppression (82). Studies have demonstrated that different types of immune cells infiltrating the tumour can indicate different prognoses in patients, and M2 macrophages have been significantly associated with worse outcomes for patients with ovarian cancer (83, 84). In the hypoxic microenvironment, ovarian cancer cells can recruit macrophages and induce their M2 transformation. Transformed macrophages likely promote the expression of miR-233 *via* an HIF-1α-dependent pathway, and miR-233 is then secreted by exosomes, which can be internalized by ovarian cancer cells. Drug resistance is promoted *via* exosomal-derived miR-233, which activates the PI3K/AKT pathway by suppressing the expression of PTEN (85). Cancer stem-like cells (CSCs) constitute a group of special cells that have self-renewal ability and are associated with chemoresistance (86). Cytokine-induced killer cells (CIKs) were recognized in the 1990s, and investigations demonstrated that CIKs may serve in a novel treatment of cancers, including ovarian cancer (87, 88). Lymphocyte function-associated antigen-1 (LFA-1) is located on the membrane of CIKs and can specifically recognize intercellular adhesion molecule-1 (ICAM-1), which is highly expressed in tumour cells, thereby mediating tumour cell death (89–91), which means that the downregulation of ICAM-1 may contribute to cancer cell protection against the killing effect. In spheroid cells, which are mainly constructed by CSCs, HIF-1α downregulates ICAM-1, shielding CSCs from the effect of cellular lysis mediated by CIK cells (92), and contributes to the progression of ovarian cancer (**Figure 3**).

### Adipocytes

Obesity has been proven to be associated with a poor prognosis in ovarian cancer (93, 94). Studies have demonstrated that adipocytes promote ovarian cancer progression (95, 96). If metastasis was a random event in ovarian cancer, then the organs in the peritoneal cavity would be equally affected by focal metastasis. However, the most common distant metastasis site is the omentum, which is primarily composed of adipocytes (97). Adipocytes secrete monocyte chemotactic protein-1 (MCP-1) to bind C-C motif chemokine receptor 2 (CCR-2) on ovarian cancer cells to activate the PI3K/AKT/mTOR pathway, thereby increasing the expression of HIF-1α, which contributes to ovarian cancer metastasis (98). During the process of adipocyte differentiation, autotaxin (ATX) is released from adipocytes and promotes the synthesis of lysophosphatidic acid (LPA) (99), which is present at a high concentration in the ascites of patients with ovarian cancer (100). Early in 2006, research showed that the PI3K/Akt/mTOR pathway may be required for LPA-induced activation of HIF-1α (101). Activation of the PI3K/AKT/mTOR/HIF-1α axis promoted the expression of Twist, a transcription factor that increases discoidin domain receptor 2 (DDR2), which is activated by collagen I (102), and then upregulates the expression of membrane type 1-matrix metallopeptidase 14 (MT1-MMP) and LOX, which is an essential factor in the invasion of ovarian cancer (103, 104) (**Figure 3**).

## Molecules Suppressed Ovarian Cancer Progression by Directly or Indirectly Downregulating HIF-1α

### Natural Compounds Extracted From Plants and Their Derivatives

**Figure 4:**
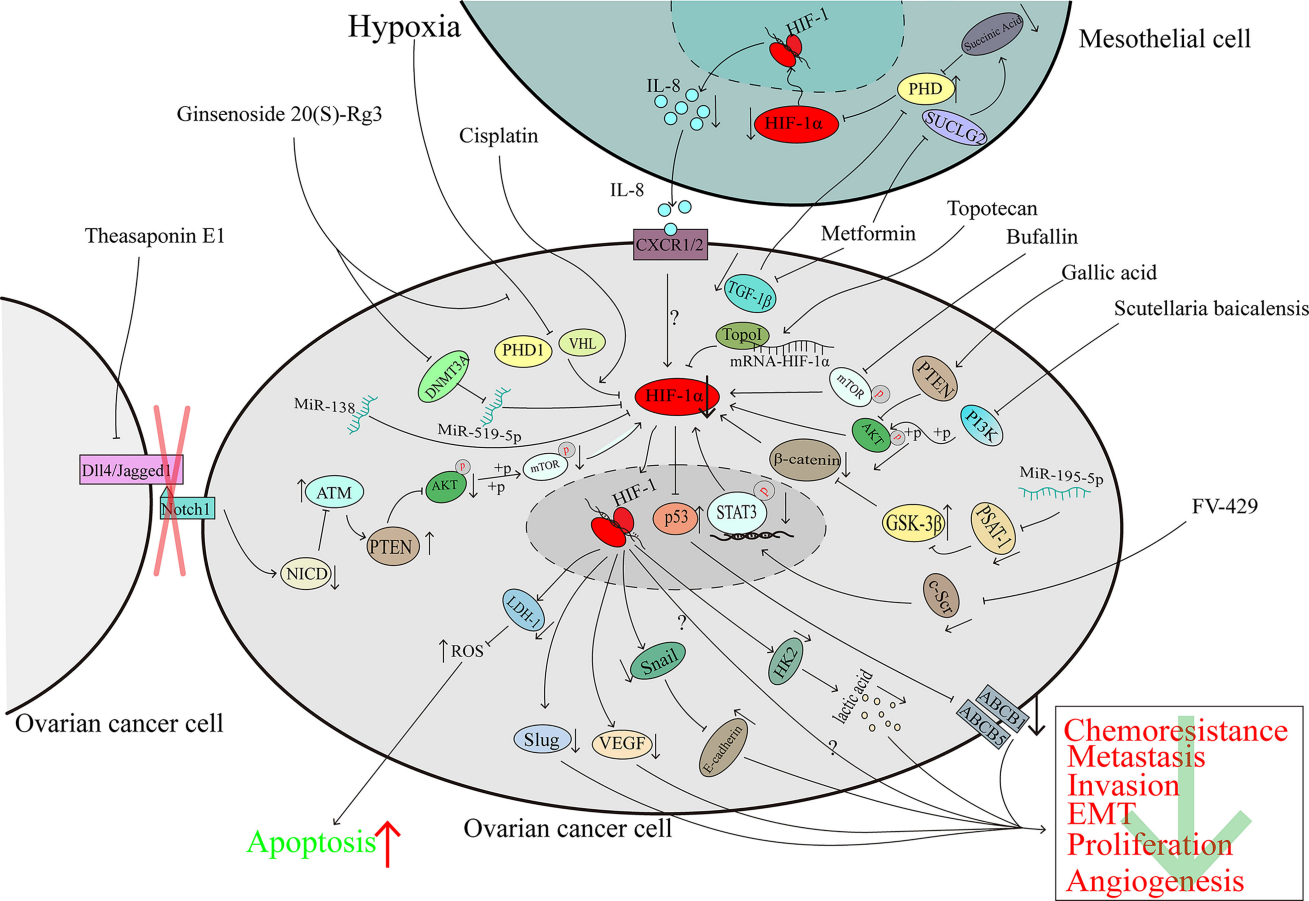
The downregulation of HIF-1α can inhibit ovarian cancer progression. The character “?” indicates that the researchers did not elaborate on the precise mechanism in their reported study.

Ginsenoside 20(S)-Rg3 is an antitumoural compound extracted from Panax ginseng which is a traditional Chinese herb (105). Ginsenoside 20(S)-Rg3 can facilitate HIF-1α degradation *via* the activation of the PHD1-VHL-ubiquitin/proteasome pathway, downregulate the expression of E-cadherin, and block the epithelial-mesenchymal transition of ovarian cancer cells *in vitro* and *in vivo* (106). Ginsenoside 20(S)-Rg3, could upregulate the expression of miR-519a-5p, which could bind to the 3’-UTR of HIF-1α mRNA, then directly downregulated the expression of HIF-1α (107). Considering that the Warburg effect plays a large role in promoting cancer progression (63), the inhibition of HIF-1α mediated by miR-519a-5p suppressed the expression of HK2, which plays an important role in the Warburg effect, and this pathway may explain, at least partly, the reason why ginsenoside 20(S)-Rg3 shows antitumoural activity ability in ovarian cancer (107) (**Figure 4**).

Topotecan (TPT) is a derivative of camptothecin which originates from the Camptotheca acuminata (108) and is used in the second-line treatment of ovarian cancer. A clinical trial demonstrated that TPT can downregulate HIF-1α in solid advanced tumours (109). In human glioma cells, TPT can downregulate HIF-1α in a topo-1-dependent manner (110). U251-HRE xenografts were treated with a low dose of daily TPT combined with bevacizumab; tumour growth was suppressed significantly, and the DAN-damage level of the two-agent treatment group was similar to that of the TPT-treatment group which indicates that the suppression of HIF-1α protein may contribute to the growth suppression (111). In ovarian cancer, TPT promotes mRNA-HIF-1α:Topo I complex formation and then hinders the translation of the HIF-1α protein (45). Because the p53 transcriptional function is eliminated when p53 binds with HIF-1α, the deletion of HIF-1α mediated by TPT can restore the function of p53, downregulate the expression of ABCB5 and ABCB1, modulate the cisplatin and paclitaxel resistance of ovarian cancer and promote apoptosis (45) (**Figure 4**).

For many years, phenolic compounds extracted from plants have been shown to play a critical role in the fight against cancer (112). In 2020, research showed that polyphenol extracts of Carya cathayensis can inhibit the proliferation of ovarian cancer and suppress VEGF expression *via* the inhibition of HIF-1α (113). However, early in 2016, gallic acid, a main polyphenolic compound of C. cathayensis, was shown to upregulate PTEN expression and suppress the phosphorylation of AKT, which led to the downregulation of HIF-1α and VEGF to hamper angiogenesis in ovarian cancer (114) (**Figure 4**).

The total extract of Scutellaria baicalensis inhibits the expression and enhances the degradation of HIF-1α *via* the inactivation of the PI3K/AKT and MEK/ERK pathways and the promotion of the proteasome and lysosome pathways, respectively. The downregulation of HIF-1α reverses the chemoresistance of ovarian cancer cells to cisplatin (115). Wogonin is a main component of S. baicalensis Georgi. It has been demonstrated that FV-429, a derivative of wogonin, has antitumoural activity (116). In hypoxic ovarian cancer cells, FV-429 can interfere with the expression and phosphorylation of c-Scr, inhibit the translocation and DNA binding activity of STAT3, and inhibit HIF-1α expression, causing the downregulation of HK2 and VEGF and enhancement of the G2/M arrest induced by paclitaxel (117) (**Figure 4**).

The total triterpenoid saponins extracted from the seeds of Camellia sinensis contribute to the antiangiogenetic effect on ovarian cancer by reducing VEGF expression in a HIF-1α-dependent manner (118). Theasaponin E1, as the main component of the C. sinensis extract (119), can reduce the expression of Dll4 and Jagged1 to inhibit the Notch1 pathway, and the Notch1 pathway is known to inactivate ATM in other studies. The activation of ATM upregulates the expression of PTEN and reduces the phosphorylation of AKT and the downstream proteins of AKT pathways, such as HIF-1α, thereby inhibiting the expression of VEGF (120, 121) (**Figure 4**).

### Compounds Extracted From Animal

Not only compounds extracted from plants, but also animal can inhibit HIF-1α expression and exhibit the ability to suppress ovarian cancer progression. Bufalin, which is obtained from the skin and parotid venom glands of toads, is a common traditional Chinese medicine. Bufalin has been proven to protect against various kinds of cancers, including ovarian cancer (122, 123). Bufalin did not affect the viability of normal ovarian epithelial cells even at doses as high as 40 μM but significantly restrained the growth of the OAW28 cell line (an ovarian epithelial carcinoma cell line). In ovarian cancer cells, bufalin could downregulation of HIF-1α *via* inhibiting the phosphorylation of mTOR and then inducing the suppression cell growth and migration (124) (**Figure 4**).

### Synthetic Drugs

Currently, cisplatin is the first-line chemotherapy drug for a variety of malignant tumours and HIF-1α is associated with cisplatin-resistance (124).However, in the cisplatin-sensitive ovarian cancer cells, cisplatin promotes HIF-1α degradation *via* the proteasome pathway, induces the downregulation of LDH-A expression, and then increases the level of reactive oxygen species (ROS) by inducing the cells to produce ATP through oxidative phosphorylation, which modulates cisplatin resistance and promotes the death of ovarian cancer cells (125–127) (**Figure 4**).

Although metformin is a common agent for diabetes treatment, a study has shown that metformin can inhibit the expression of HIF-1α and the growth of ovarian cancer cells (128). As previously noted, mesothelial cells in the tumour microenvironment of ovarian cancer play crucial roles in tumour progression (81). In addition to its influence on cancer cells alone, in mesothelial cells, metformin induces the expression of the tricarboxylic acid (TCA) enzyme succinyl CoA ligase (SUCLG2), leading to metabolic reprogramming and reducing the production of succinic acid. As an inhibitor of PDH, succinic acid causes HIF-1α degradation. In addition, metformin induces the downregulation of TGF-1β in ovarian cancer cells, and the reduction in secreted TGF-1β restores PDH activity, leading to increases in HIF-1α degradation. In summary, the reduced expression of HIF-1α results in the downregulation of IL-8 and hinders the invasion of ovarian cancer cells (129). Considering that IL-8 can promote ovarian cancer progression through several pathways (130–132), it is recommended that further investigation be directed towards the pathways by which metformin mediates its effects on ovarian cancer (129) (**Figure 4**).

SC-144, a novel synthetic agent, can target gp130 and kill ovarian cancer cells (133). A genome-wide bromouridine sequencing (Bru-seq) analysis showed that longer exposure to SC144 led to lower HIF-1α expression but a higher hypoxia-inducible factor antisense (HIF-1α-AS) level (134). Considering that HIF-1α-AS downregulates the expression of HIF-1α (135) and because HIF-1α plays a role in the progression of cancer, we speculate that SC-144 inhibits the proliferation of ovarian cancer, at least to some extent, *via* the HIF-1α-AS/HIF-1α axis. However, the function of HIF-1α-AS in malignant tumours is complicated (136, 137), and data on the role of HIF-1α-AS in ovarian cancer have not been reported. Hence, future investigation into the function of the HIF-1α-AS/HIF-1α axis in ovarian cancer is recommended (**Figure 4**).

### Noncoding RNAs

MiRNAs belong to the family of noncoding RNAs, and some miRNAs serve as a sponge to regulate the expression of genes and influence the development of cancer. Transfection of miR-195-5p can inhibit PSAT1 directly because this miRNA interacts with the 3’-UTR of PSAT1 mRNA, thus suppressing the phosphorylation of β-catenin and GSK3β, downregulating the expression of HIF-1α and VEGF, inducing apoptosis and reducing cisplatin chemoresistance (138). MiR-138 is downregulated in ovarian cancer, especially in invasive cell sublines, and acts as a cancer suppressor. Overexpression of miR-138 downregulates HIF-1α expression and induces the inhibition of Slug (139), which is associated with ovarian cancer metastasis (140) (**Figure 4**).

## Drug Clinical Trials of the HIF-1α Inhibitors in Solid Malignant Tumours

### 2-Methoxyestradiol (2ME2)

2ME2 is a derivative of estradiol and has been proven to downregulate HIF-1α at the posttranscriptional level (153). In 2009, a phase II study of 2ME2 administered at a dose of 1000 mg four times per day in recurrent, platinum-resistant ovarian cancer patients reported that no objective response was observed in the study, but 7 out of 18 patients had stable disease and 2 of them had stable disease for more than 12 months (142). In taxane-refractory, metastatic castration-resistant prostate cancer patients, 2ME2 did not benefit patients with a poor PFS at 6 months rate (only 5.35%) (141). In another phase II clinical trial, patients were divided into two arms (arm A,2ME2 alone, n=10; arm B, 2ME2 combined with sunitinib malate, n=7). However, owing to intolerance toxicities that may be caused by a high dose of 2ME2 (1500 mg three times per day), 6 patients were required to quite the study, and no objective responses were observed in the two arms (143) (**Table 2**).

**Table 2:** Drug clinical trials with HIF-1α inhibitors.

Agent	Disease/cases	Combined with other agent(s)	Outcome	Ref.
2ME2	Prostate cancer/21	none	PFS-6 mo:5.35%	(141)
	Ovarian cancer/18	none	ORR:0; SD:38.89%	(142)
	Renal cell cancer/12	Arm A: +sunitinib malate	SD:57%	(143)
		Arm B: +none	SD:60%	
Tanespimycin	Renal cell cancer/20	none	CR or PR:0; SD:70%	(144)
	Prostate cancer/15	none	PSA PFS:1.8 mo	(145)
Vorinostat	Renal cell cancer/33	bevacizumab	OR:18%; PFS-6 mo:48%; PFS:5.7 mo; OS: 13.9 mo	(146)
	Melanoma/32	none	PR:6%; SD:50%	(147)
EZN-2968	Refractory advanced solid tumour/10	none	Decreased HIF-1α at mRNA level:5;	(148)
			Decreased HIF-1α at protein level:3	
	Hepatocellular cancer/9	none	Decreased HIF-1α at mRNA level in patients had SD and PR	(149)
			SD:11.1%	
			PR:11.1%	
EZN-2208	Colorectal cancer/211	Arm A: +none	No radiographic response were observed	(150)
		Arm B: +cetuximab	OR:8%; PFS: 4.9 mo; OS:9.8 months; PFS-6 mo:37%	
		Arm C: irinotecan+cetuximab	OR:5%; PFS: 3.7 mo; OS:9.1 months; PFS-6 mo:29%	
CRXL101	Renal cell cancer/111	Arm A: bevacizumab	PFS: 3.7 mo	(151)
		Arm B: other agents	PFS: 3.9 mo	
	Ovarian cancer/63	Arm A: none	ORR: 11%; PFS: 4.5 mo	(152)
		Arm B: +bevacizumab	ORR: 18%; PFS: 6.5 mo	

PFS, progression-free survival; mo, months; PFS-6 mo, progression-free survival at 6 months; ORR, overall response rate; CR, complete response; PR, partial response.

### Tanespimycin

Heat-shock protein 90 could stabilize the HIF-1α protein by inhibiting the ubiquitination and proteasomal degradation of HIF-1α (154). Tanespimycin is a heat-shock protein 90 inhibitor (155). In 2006, 20 renal cell cancer (RCC) patients were enrolled in a phase II study that focused on the efficacy and toxicities of tanespimycin. Five of eight papillary renal cell cancer patients and 9 of 12 patients had stable disease, but none of them achieved complete or partial response. Thirty percent of patients required a reduced dose because of toxicities (144). In hormone-refractory metastatic prostate cancer patients, none achieved a PSA response and the 6-month OS rate was 71% (145) (**Table 2**).

### Vorinostat

Vorinostat inhibits HIF-1α protein expression at the translational level (156).In 2014, a total of 32 melanoma patients were given vorinostat, 18 of whom had stable disease with a median PFS of 5 months or partial response. For the patients with partial response, one remained for 7 cycles, and the other remained for 5 cycles; each cycle lasted 28 days. In addition, two patients who had stable disease had dramatic responses (33-50% shrinkage), which lasted only approximately two months. The time is too short to be confirmed as a response (147). In 33 clear-cell renal cell carcinoma patients who received vorinostat combined with bavacizumab, the PFS at 6 months was 48%. The median PFS was 5.7 months, while the OS was 13.9 months. Six objective responses were observed, and 19 patients had stable disease (146) (**Table 2**).

### EZN-2968

EZN-2968 is an RNA antagonist that can specifically bind to and inhibit the expression of HIF-1α mRNA to downregulate HIF-1α protein expression in cancer cells (157). In a pilot trial of patients with refractory advanced solid tumours, EZN-2968 could downregulated HIF-1α expression at the mRNA (5/6) and protein (3/5) levels in some patients (148). In a phase Ib trial, 2 of 9 advanced hepatocellular cancers had a partial response or stable disease, and the HIF-1α mRNA was downregulated in the cancer tissue (149) (**Table 2**).

### EZN-2208

EZN-2208 is a soluble derivative of SN-38, which is an active metabolite of irinotecan (158).EZN-2208 could inhibit the expression of HIF-1α mRNA and protein, which is superior to irinotecan, thus controlling the angiogenic response (159). A total of 211 advanced colorectal cancer patients were enrolled in a phase II study, and were divided into 3 arms(arm A: EZN-2208, for KRAS-mutant patients; arm B: EZN-2208+cetuximab, for KRAS-wild-type patients; arm C: irinotecan+cetuximab, for KRAS-Wild type patients). When comparing the OR, OS, PFS and PFS at 6 months rate between arm B and arm C, arm B showed slightly superior efficacy. However, there was no statistically significant difference between these two arms (150) (**Table 2**).

### CRLX101

Antiangiogenic therapy induced increased HIF-1α expression, and CRLX101 reduced the HIF-1α expression when combined with bevacizumab in animal models (160). In a phase II study of 63 recurrent ovarian cancer patients, 29 patients who received single agent CRLX101 had an overall response rate (ORR) of 11%. When 34 patients were treated CRLX101 combined with bavacizumab, the ORR was increased to 18% (152). However, in another phase II study of 111 advanced renal cell cancer patients, CRLX101 combined with bevacizumab did not show any added benefit to patients compared with standard treatment (151) (**Table 2**).

## Conclusion and Future Prospects

**Figure 5:**
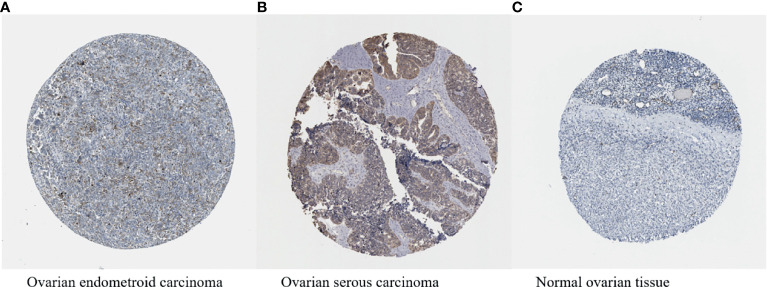
**(A–C)** HIF-1α expression in ovarian cancer (available from http://v13.proteinatlas.org/ENSG00000100644-HIF1A/cancer/tissue/ovarian+cancer#img) is higher compared with that in normal ovarian tissue (available from https://www.proteinatlas.org/ENSG00000100644-HIF1A/tissue/ovary#img). The brown staining indicates the presence of HIF-1α.

HIF-1α has been proven to be overexpressed in more than 70% of human cancers, including ovarian cancer (**Figures 5A**–**C**) (161, 162), and occupies a central position in multiple pathways of ovarian cancer. HIF-1α acts as a transcription factor to regulate a variety of proteins, thereby promoting the development of ovarian tumours. In the ovarian cancer microenvironment, various factors can also regulate the expression of HIF-1α expression in nontumour cells and affect the malignant biological properties of tumour cells.

On the basis of the proposed concept of precision medicine, targeted drugs developed based on tumour characteristics have emerged in an endless stream, and among these drugs, antiangiogenic agents mainly target VEGF, thereby inhibiting a series of pathophysiological processes regulated by VEGF and benefiting patients with tumours such as ovarian or breast cancer (163, 164). Because VEGF is a downstream gene of HIF-1α, VEGF expression is decreased when the expression or function of HIF-1α is inhibited (55, 113, 114, 118), and HIF-1α can regulate the expression of other genes that promote tumour progression. Therefore, we concluded that targeting HIF-1α may effectively inhibit tumour development. Studies have shown that, regardless of whether a therapy is based on monomeric components extracted from plants or classic drugs that have been clinically used in cancer treatment for many years, a therapy can inhibit ovarian cancer progression after directly or indirectly inhibiting HIF-1α.

Recently, clinical trials have been conducted to evaluated drugs that could modulate HIF-1α expression in many kinds of solid tumours. However, the efficacy has been limited and varied in these trials, and only EZN-2968 could combined with the HIF-1α mRNA to regulate HIF-1α protein expression. The remaining drugs all regulated HIF-1α indirectly. It is suggested to explore new drugs that could interact with HIF-1α protein directly. In addition, almost all of the drugs were taken orally. Hypoxia occurs in tumours and is associated with the newly formed abnormal microvessels (165), and chemotherapy drugs cannot reach the tumour site due to the high interstitial fluid pressure caused by the abnormal microvessels (166). This situation means that HIF-1α inhibitors may not influence the cells that produce HIF-1α. It is not only recommended to develop new agents that target HIF-1α directly but also attach importance to the delivery method of drugs so that ideal drug concentrations can be reached. In a clinical trial of ovarian cancer, the ORR and PFS were superior in the bevacizumab+HIF-1α inhibitor group to the HIF-1α inhibitor group (152). We may infer that in the application of HIF-1α inhibitor to treat ovarian cancer, it is better to combine HIF-1α inhibitor with other agents.

In view of the tremendous heterogeneity between different types of tumours, the unsatisfactory results found in cancers now do not necessarily indicate a failure of these kinds of agents in the future. Clinical trials have shown that combining the HIF-1α inhibitors and bevacizumab may benefit ovarian cancer patients (152). Further exploration into the efficacy of HIF-1α inhibitors in ovarian cancers is necessary. In addition, since HIF-1α is a transcription factor that facilitates both malignant and normal cell adaptation to hypoxic stress in the internal environment, it is particularly important to design drugs targeting only HIF-1α expressed in tumours to reduce the adverse effects.

## Author Contributions

HZ and XW contributed to the conception, design and drafting of the manuscript. Z-wD, T-mX, and X-jW contributed to data collection and drafting the manuscript. WL and J-lG prepared the figures. JL prepared the tables. All authors approved the final version for submission. HZ oversaw the study.

## Funding

This study was supported by the Health Special Project Founds of Jilin Province (Grant No. 2020SCZT042), and the Jilin Science and Technology Funds, China (Grant No. 20210204025YY).

## Conflict of Interest

The authors declare that the research was conducted in the absence of any commercial or financial relationships that could be construed as a potential conflict of interest.

## Publisher’s Note

All claims expressed in this article are solely those of the authors and do not necessarily represent those of their affiliated organizations, or those of the publisher, the editors and the reviewers. Any product that may be evaluated in this article, or claim that may be made by its manufacturer, is not guaranteed or endorsed by the publisher.

## Abbreviations

GLUT-1, glucose transporter 1; LDH-1, lactate dehydrogenase 1; MCT4, monocarboxylate transporter 4; SIK2, salt-inducible kinase 2; PI3K, phosphatidylinositol 3 kinase; AKT, protein kinase B; HK2, hexokinase 2; PFKL, phosphofructokinase, liver type; GEHT1, gastric carcinoma high expressed transcript 1; BAX, B-cell lymphoma-2 associated X; mTOR, mammalian target of rapamycin; JAK2, Janus kinase 2; STAT3, signal transducer and activator of transcription 3; CDKN2B-AS1, antisense noncoding RNA in the INK4 locus; VEGF, vascular endothelial growth factor; DSCR8, Down syndrome critical region 8; COL1A1, collagen, type I, alpha 1; PTEN, phosphatase and tensin homologue deleted on chromosome ten; ABCB5, ATP-binding cassette, sub-family B, member 5; ABCB1, ATP-binding cassette, sub-family B, member 1; MEK, mitogen-activated protein kinase kinase; ERK, extracellular regulated protein kinases; Dll4, delta-like ligand 4; ATM, ataxia telangiectasia mutated protein; TGF-1β, transforming growth factor beta 1; PSAT1, phosphoserine aminotransferase 1; GSK3β, glycogen synthase kinase 3 beta.
